# Characterization of the T-cell receptor repertoire associated with lymph node metastasis in colorectal cancer

**DOI:** 10.3389/fonc.2024.1354533

**Published:** 2024-11-12

**Authors:** Ya’nan Zhen, Hong Wang, Runze Jiang, Fang Wang, Cunbao Chen, Zhongfa Xu, Ruixue Xiao

**Affiliations:** ^1^ Department of Gastrointestinal Surgery, Shandong Provincial Third Hospital, Shandong University, Jinan, Shandong, China; ^2^ Jinan Biomedical Industry Academy of Shandong First Medical University, Jinan, Shandong, China; ^3^ Department of Gastrointestinal Surgery, The Third Affiliated Hospital of Shandong First Medical University, Jinan, Shandong, China; ^4^ Gastroenterology Research Institute and Clinical Center, Shandong First Medical University, Jinan, Shandong, China; ^5^ Department of Pathology, Shandong Provincial Third Hospital, Shandong University, Jinan, Shandong, China

**Keywords:** colorectal cancer, lymph node metastasis, T-cell receptor repertoire, TCR sequencing, biomarkers

## Abstract

**Purpose:**

Colorectal cancer (CRC) is a leading cause of cancer-related mortality worldwide, with lymph node (LN) metastasis playing a pivotal role in disease progression. This study aimed to explore the T-cell receptor (TCR) repertoire among CRC patients, distinguishing those with LN metastasis from those without, in order to uncover potential biomarkers for predicting metastasis.

**Methods:**

We analyzed the TCR repertoire in CRC patients with and without LN metastasis. A classification model utilizing random forest analysis was developed to assess the predictive potential of the TCR repertoire.

**Results:**

The findings demonstrated a significant increase in the number of V-J combinations and immune CDR3 sequences in patients with LN metastasis compared to the control group. The classification model achieved high accuracy in differentiating patients with LN metastasis, with AUC values ranging from 0.514 to 0.794. Specific V-J combinations and CDR3 sequences were identified as significant predictors of the model’s predictive accuracy.

**Conclusion:**

These results suggest that the TCR repertoire is altered in CRC patients exhibiting LN metastasis, potentially influencing disease progression. This study highlights the importance of TCR repertoire analysis as a non-invasive biomarker for predicting LN metastasis in CRC patients.

## Introduction

In 2020, colorectal cancer (CRC) was estimated to account for nearly one in ten cancer cases and deaths, with over 1.9 million new cases and 935,000 fatalities worldwide (1). In terms of incidence rates, CRC ranks as the second most common cancer and is the third leading cause of cancer-related deaths. Despite significant advancements in screening and treatment methods, CRC continues to pose a formidable challenge due to its complex molecular mechanisms and inherent heterogeneity (2).

The lymph node (LN) system serves as the primary immunological defense against the spread of tumor cells, and the infiltration of tumor cells in LNs is a strong predictor of poor prognosis (3). The predominant route for CRC metastasis is through the LN system, with early LN involvement being a common occurrence. The presence of LN metastasis not only indicates that the tumor has spread beyond its original site but also suggests an increased risk of extensive systemic dissemination. Consequently, LN involvement is a critical determinant in the American Joint Committee on Cancer’s TNM staging system, which is currently the most reliable prognostic classification system guiding treatment strategies (4). TNM staging classifies a patient as having a pathological stage of III or higher when LN metastasis is evident, necessitating postoperative combination adjuvant treatment.

LN yield is associated with a robust immune microenvironment (5) and patients who undergo extensive LN dissection have a higher chance of survival (6, 7). Recent studies have found that LN-high cancer exhibits less distant metastasis, whereas LN-low carcinoma shows relatively more distant metastasis. A strong immune response may help to prevent distant metastasis but may also result in a greater LN harvest (5). Regional LNs are thought to play essential roles in antitumor immunity, as T cells are generally primed by antigen-presenting cells that capture tumor antigens in tumor tissues and migrate to regional LNs. Typically, T lymphocytes are activated in LNs before infiltrating tumor tissues; thus, localized LNs frequently play a key role in the development of antitumor immunity (8, 9).

LN metastasis signifies not only the tumor cells’ ability to invade surrounding tissues but also often becomes a crucial site for the interaction between immune cells and tumor cells. The interplay between tumor cells and immune cells significantly impacts the further dissemination of the tumor and the prognosis of the patient (10). T cells recognize and respond to tumor antigens through their surface TCRs, playing a vital role in immune surveillance and attacks. In CRC, LN metastasis correlates significantly with the level of T-cell infiltration in the tumor microenvironment, a relationship that may reflect the complexity of the tumor immune microenvironment and its dynamic changes during tumor progression (11). In patients with CRC and LN metastasis, specific TCR clonotypes correlate with the level of immune cell infiltration in the tumor microenvironment. These TCR clonotypes may be involved in the process of tumor immune evasion or reflect the complex interactions between immune and tumor cells within the tumor microenvironment (12).

T cells play a critical and indispensable role in the adaptive immune response against cancer cells, as they possess the extraordinary ability to recognize and eliminate malignant cells (13, 14). TCRs are essential for initiating T-cell-mediated antitumor functions because they trigger immune responses, including cytokine production, that impede tumor growth (14, 15). Additionally, TCRs stimulate cytotoxic activity against cancer cells and recruit other immune cells to the tumor site (14–16). Each TCR consists of an alpha chain and a beta chain. The beta chain, which is encoded by the TCRB gene, is responsible for conferring antigen-specific binding to the TCR (17). This broad spectrum gives T cells the potential to recognize and bind a wide range of tumor-specific antigens (18). This unique ability makes T cells a key component of immune surveillance and enables them to actively participate in the targeted destruction of cancer cells.

Past studies have extensively examined the transcriptome of peripheral blood and tumor tissue samples, identifying the upregulation of immune-related genes as a common feature (19–21). However, determining whether a specific upregulated gene serves as a biomarker for LN metastasis in CRC remains challenging. This difficulty may arise from differences among various research designs, patient groups, and analytical techniques, among other potential causes. Due to the heterogeneity of the transcriptome, functional dysregulation of immune cells may only occur in a subset of cases that may be overlooked by whole-genome sequencing protocols (22). The TCR repertoire is shaped by antigen engagement and provides disease-related TCR clonotypes that evolve as the disease progresses. Therefore, highly diverse TCR repertoires, generated by genomic rearrangement of the variable (V), diversity (D), and joining (J) regions, are crucial for mediating adaptive immunity in humans (23). Our study reveals the association between LN metastasis in CRC and the diversity of the TCR repertoire, providing a new perspective for understanding the interactions within the tumor immune microenvironment and potentially aiding in the development of novel immunotherapeutic strategies.

## Materials and methods

### Study participants

Sixty fresh tumor tissues were collected from CRC patients who underwent surgical resection between 2018 and 2022 at the Third Affiliated Hospital of Shandong First Medical University (Affiliated Hospital of Shandong Academy of Medical Sciences). The inclusion criteria for this study were as follows: (1) histopathologically confirmed primary adenocarcinoma of the colon or rectum, and (2) availability of complete clinical data. Patients were excluded from the study if they: (1) had received radiotherapy, chemotherapy, immunotherapy, or targeted therapy prior to surgery, (2) had a history of other malignant tumors, or (3) suffered from autoimmune diseases or chronic conditions. Overall, we obtained 40 samples showing LN metastasis and 20 samples showing non-LN metastasis. This study was approved by the Ethics Committee of the Third Affiliated Hospital of Shandong First Medical University (Approval No. FY2021018). All participants provided written informed consent prior to participation in this study. Tumor samples were obtained immediately after surgical resection, washed in an ice-cold saline solution, and then frozen in liquid nitrogen until further analysis.

### Sample processing

Tumor tissue samples were collected from patients and immediately placed into EDTA vacutainer tubes, each containing a volume exceeding 2 mL. The samples were promptly transported on ice to the laboratory, where they were processed for RNA extraction within a maximum of 24 hours to maintain RNA integrity. Total RNA extraction was performed using the RNAsimple Total RNA Kit (DP419, Tiangen Biotech, Beijing, China), following the manufacturer’s protocol. The concentrations of the extracted total RNA were measured using a NanoDrop ND-2000 spectrophotometer (Thermo Scientific, UK). Absorbance ratios at 260/280 nm were used to evaluate RNA purity, with values between 1.8 and 2.0 considered acceptable, indicating high-quality RNA suitable for downstream applications. Total RNA from the samples was reverse transcribed into complementary DNA (cDNA) using reverse transcriptase enzymes provided in the kit. cDNA was allowing for subsequent amplification and sequencing.

### TCR sequencing

cDNA of all potential rearranged T cell receptor (TCR) β-chain sequences were carried out using the Immune Repertoire Library Preparation Kits (Geneway, Jinan, China). The procedure was conducted following a previously described protocol (24). Multiplex PCR was then employed to amplify these cDNA, targeting all possible rearranged TCR β-chain sequences. This amplification involved a combination of multiple primers designed to span various V (variable), D (diversity), and J (joining) gene segments of the TCR β-chain locus. Before sequencing, the TCR libraries were quantified and assessed for size distribution using an Agilent Bioanalyzer (Agilent Technologies, USA). The amplified TCR libraries were sequenced on a DNBSEQ-T7 platform (MGI, Shenzhen, China). This high-throughput sequencing system generated paired-end reads of 150 base pairs (bp) in length.

### Preprocessing of sequencing data

All sequencing data were stored in FASTQ format, and raw reads were demultiplexed according to the sequences of index primers corresponding to different samples. Next, low-quality sequences were discarded for quality control. A Q30 score of ≥90% was considered as benchmark for high-quality sequencing. And the size of the data volume and the error rate of each sample are also considered. The remaining sequences were mapped onto the V, D, and J gene segments of the TCR β-chain using MiXCR version 3.0.6 with default parameters for sequencing alignment and clonotype assembly (25). TCR reference gene data were downloaded from the IMGT database (http://www.imgt.org/vquest/refseqh.html). Finally, the frequency of each TCR β clonotype was converted into rpm (reads per million) values for standardization. The raw datasets generated and analyzed during this study are available in the NCBI-SRA repository, accessible at the Sequence Read Archive of the National Center for Biotechnology Information (NCBI, PRJNA1049886) https://www.ncbi.nlm.nih.gov/search/all/?term=PRJNA1049886.

### Statistical analysis and classification modeling

R version 4.1.0 (http://www.R-project.org) was used for all data analyses and figure plotting. Continuous data are presented as mean ± standard deviation, while categorical variables are presented as numbers and percentages. For continuous variables, a student’s t-test was used to compare statistical differences between groups. Fisher’s exact tests were employed to examine categorical variables. A p-value of < 0.05 was considered statistically significant. Classifiers for predicting LN metastasis were built by R package “random Forest”. And all relevant data was processing in R.

## Results

### Elucidation of enrolled patients

In the cohort of 60 enrolled patients, there were 34 males and 26 females, with an age range of 34 to 86 years and a median age of 63 years. A history of smoking was present in 17 cases, and a history of alcohol consumption was noted in 20 cases. Colon cancer was diagnosed in 28 cases, while rectal cancer was diagnosed in 32 cases. According to the TNM staging of colorectal cancer (AJCC 8th edition, 2017), stages I-II were represented by 40 cases, and stages III-IV by 20 cases. The patient cohort was divided into those exhibiting LN metastasis (n = 20) and those without LN metastasis (n = 40). We aimed to comprehensively analyze the impact of LN metastasis on the characteristics of the TCR repertoire. The clinicopathological characteristics of the included patients are summarized in **Table 1**. We found no statistically significant differences in any demographic variable between samples with and without LN metastasis. Furthermore, after comparing the maximal tumor diameter relative to actual tumor size, no appreciable differences were observed. However, a substantial disparity in the distribution of TNM stages between the two groups was noted.

**Table 1:** Clinicopathological characteristics of patients.

Clinical Characteristic	Lymph Node Metastasis (n = 20)	non-Lymph Node Metastasis(n = 40)	Fisher P value
Demographic variable			
Age ≤ 50, years, n (%)	4 (20.0)	7 (17.5)	1
Age > 50, years, n (%)	16 (80.0)	33 (82.5)	1
Sex, male, n (%)	12 (60.0)	22 (55.0)	0.7867
Sex, female, n (%)	8 (40.0)	18 (45.0)	0.7867
Smoking, yes, n (%)	9 (45.0)	8 (20.0)	0.0673
Drinking, yes, n (%)	8 (40.0)	12 (30.0)	0.5629
Symptoms			
Maximum tumor diameter ≥ 5 cm, n (%)	13 (65.0)	21 (52.5)	0.4162
Maximum tumor diameter < 5 cm, n (%)	7 (35.0)	19 (47.5)	0.4162
TNM Stage I and II, n (%)	0	40 (100.0)	2.39E-16
TNM Stage III and IV, n (%)	20 (100.0)	0	2.39E-16

### Differences in quantity characteristics between TCR repertoires

**Figure 1:**
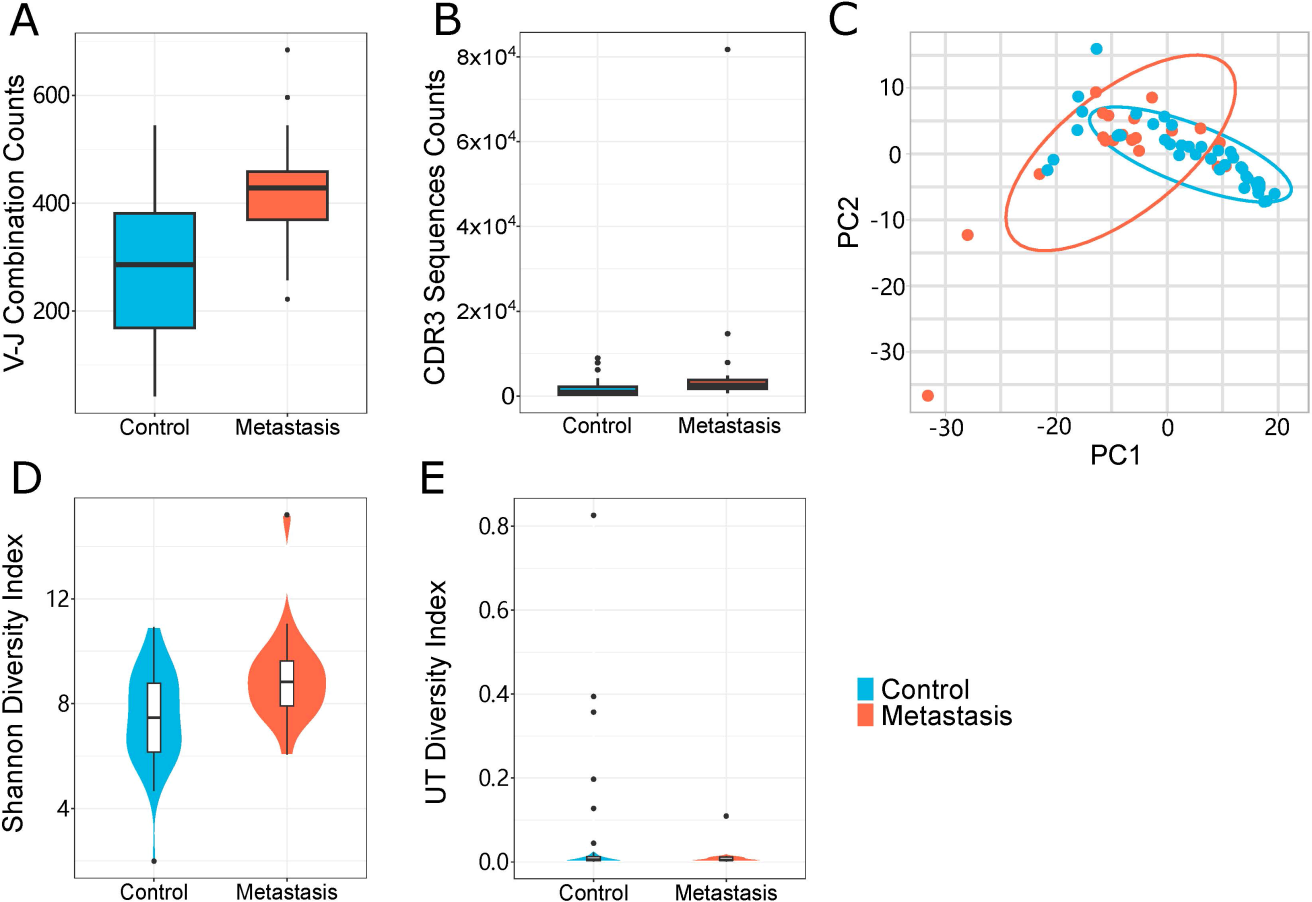
Properties of the TCR repertoire. **(A, B)** TCR sequencing quantity features of the LN metastasis and control groups. **(A)** The number of unique V-J combinations (P value = 0.0002) **(B)** Counts of unique CDR3 sequences (P value = 0.05). **(C)** A principal component analysis of the LN metastasis group and the control group. Each dot represented one sample and the ellipse indicated approximate range of the cohort sample. **(D, E)** TCR sequencing diversity features of the LN metastasis and control groups. **(D)** Shannon Diversity Index (P value = 0.0037), **(E)** UT Index (P value = 0.05). The violin chart and box plot depict data distributions that include the minimum, first quartile, median, third quartile, and maximum, with red representing the LN metastasis group and blue representing the control group. All statistical methods for these figures were Student’s t-test.

We performed TCR repertoire sequencing on tumor tissue samples to assess differences between CRC patients with and without LN metastasis. We identified the transcriptional levels of various V-J combinations and immune CDR3 sequences. V-J combinations denotes the landscape of immunization categories and immune CDR3 sequences denotes a more nuanced breakdown of immunization categories. Patients with LN metastasis exhibited a higher number of V-J combinations and immune CDR3 sequences compared to controls; these differences were statistically significant (**Figures 1A, B**). We also observed a marked increase in immunological activity during LN metastasis, evidenced by a higher number of immunological elements. By comparing colorectal cancer patients with and without LN metastasis, we identified a common immunological predisposition for LN metastasis.

### Clustering patterns base on TCR repertoire

We conducted principal component analysis (PCA) on the frequency profile of the V-J combinations. The LN metastasis and non-LN metastatic groups exhibited distinct clustering patterns (**Figure 1C**). Both groups displayed a wide range of immune defense traits, with LN metastasis showing more intricate immunological specificity. TCR expression patterns were examined to assess their relationship to systemic immune responses. The pattern base on TCR repertoire of each sample divided the two groups, which can be used as a potential means of differentiation for LN metastasis.

### Differences in diversity between LN and non-LN metastasis samples

To evaluate the diversity of TCR clonotypes in each sample, we calculated the Shannon diversity and UT diversity indices, which could describe diversity in different dimensions. The Shannon index focuses on overall diversity (both richness and evenness), while the UT index emphasizes dominance or typicality. Relative to the control group, samples from the LN metastasis group demonstrated greater Shannon diversity indices (**Figure 1D**). Additionally, the control group exhibited higher UT diversity indices than those from the LN metastasis group (**Figure 1E**). These results indicated differences in the TCR profiles of the LN metastasis and control groups, suggesting that the LN metastasis group possesses a greater quantity and variety of immune responses.

### Characteristics of the TCR repertoire

**Figure 2:**
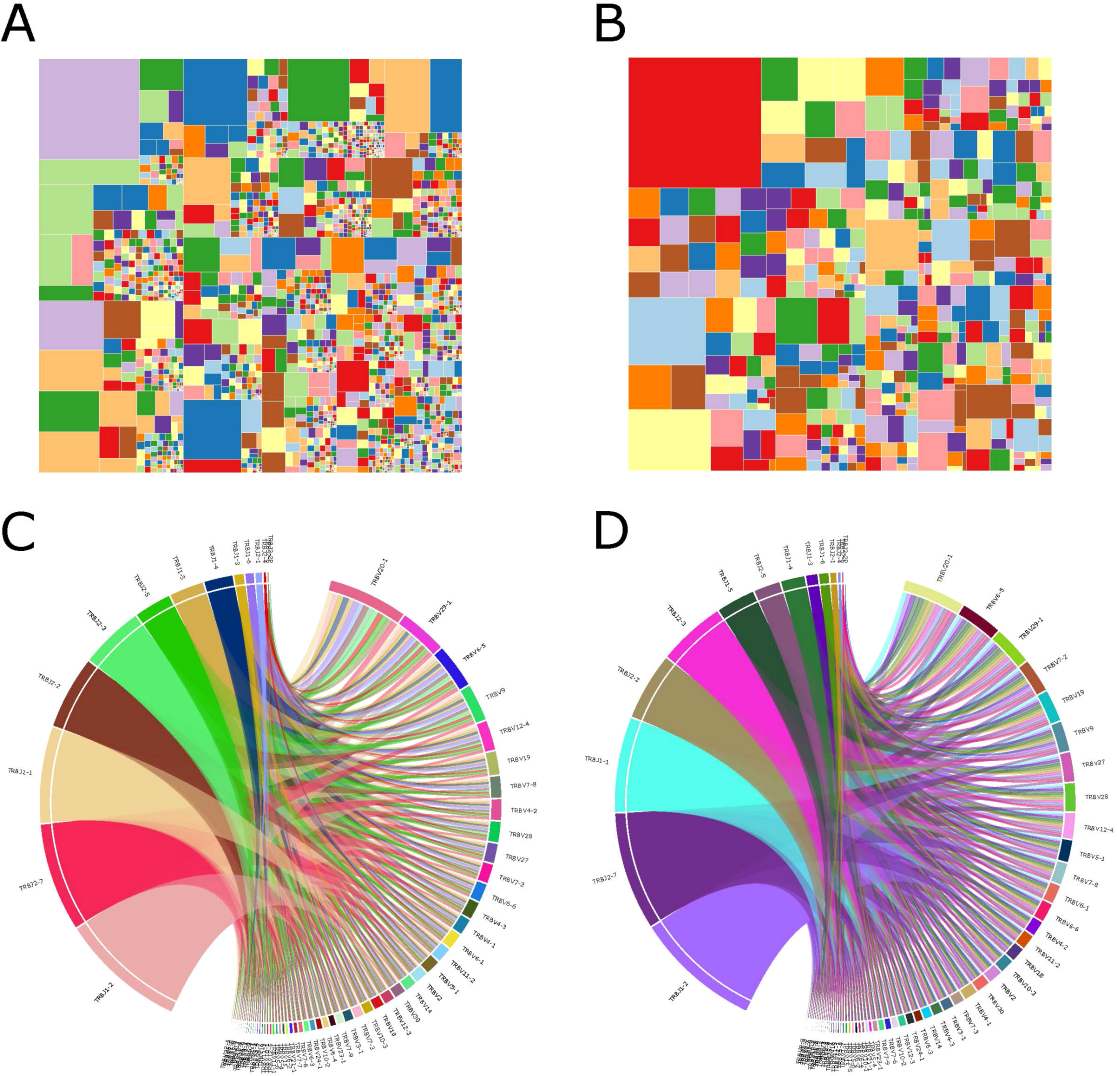
Immunological characteristics of samples. **(A, B)** Treemap showing the abundances of module CDR3 sequences of samples from the LN metastasis group **(A)** and the control group **(B)**. **(C, D)** V-J gene combinations of samples from the LN metastasis group **(C)** and the control group **(D)** are shown as a circular figure. Left and right parts represented J gene and V gene respectively. Each breadth showed combination of specific V and J genes. Colors in these two figures were randomly.

We performed a characteristics analysis on all samples to determine the specificity of immune sequence abundance in LN metastasis patients relative to non-LN metastatic controls. We discovered a high abundance of specific sequences in both groups (**Figures 2A, B**), illustrating that sequence diversity was higher in LN metastasis patients than in control samples. The overall distribution showed that LN metastasis patients showed more immune CDR3 sequence in V-J combinations. And most V-J combinations could be found representative immune CDR3 sequences. These phenomena indicated that LN metastasis patients showed a broader spectrum of immune resistance compared to control. Meanwhile, LN metastasis patients had more specific immune predisposition than control patients. Circumferential plots demonstrated that the average frequencies of specific V/J gene combinations were similar in both groups (**Figures 2C, D**). The relative frequency of V or J genes is indicated by the length of the corresponding sector, while the relative usage frequency of V/J combinations is indicated by the breadth of the link between the V and J genes. Our results indicate a significant disruption of the immunological milieu caused by specific, highly abundant sequences that impacted diversity.

### Specificity of V-J combinations and LN metastasis in CRC patients

**Figure 3:**
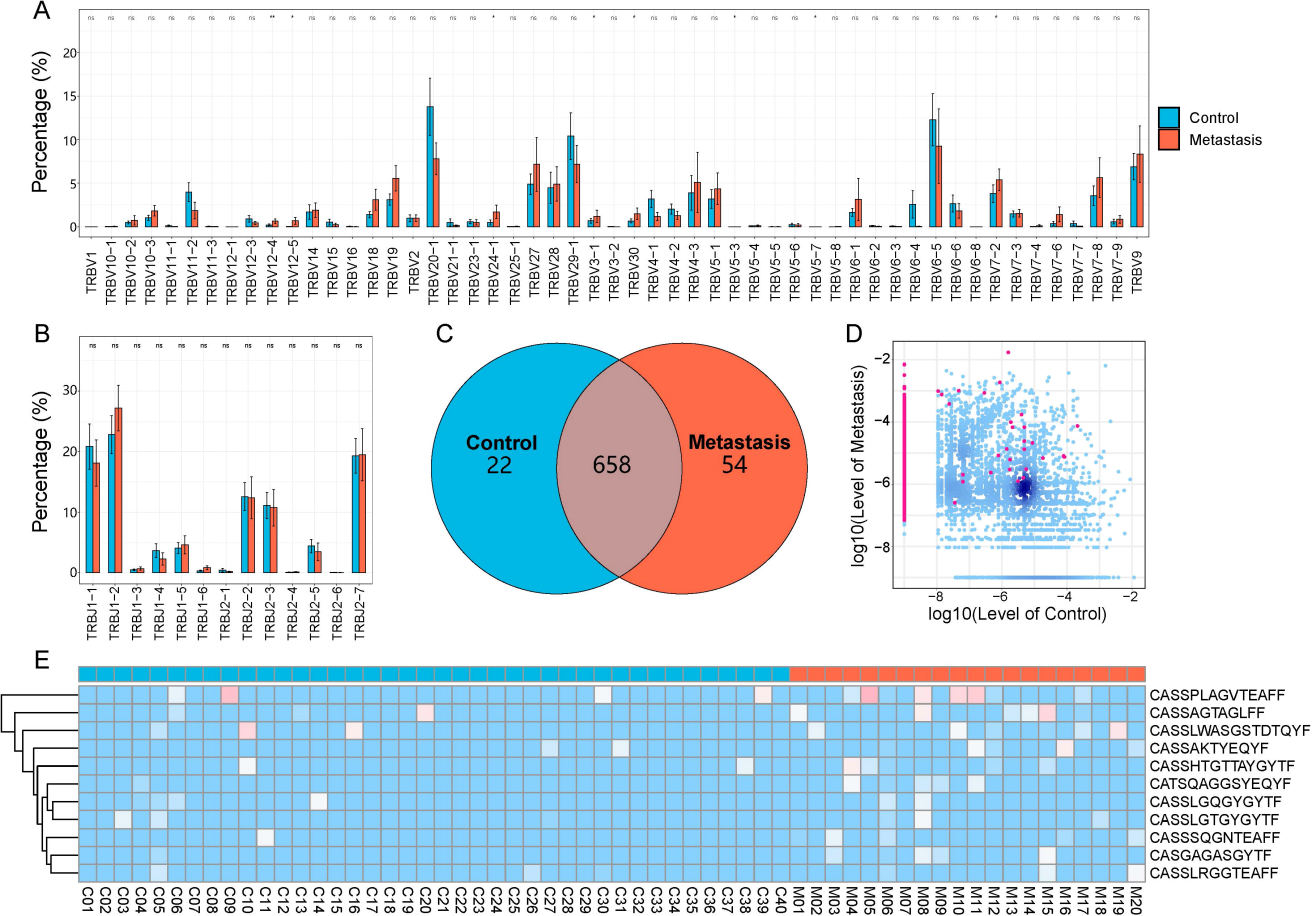
CDR3 sequences and V and J gene segments are used differently in the LN metastasis and control groups. **(A)** V gene usage. **(B)** J gene usage. The LN metastatic group is represented in red, and the control group is represented in blue. All statistical methods for these two figures were Student’s t-test. One asterisk indicated a p-value less than 0.05 and two asterisks indicated a p-value less than 0.01. **(C)** The Venn diagram depicts shared or unique V-J combinations discovered by two groups. **(D)** Each dot in the scatterplot represents a CDR3 sequence, with red denoting a substantial difference in the relative abundance of the two groups and blue denoting no difference. **(E)** Heatmap displaying differentially expressed amino acid clones. Group information was displayed in the first line according to the color feature. Blue blocks represented low abundance and red blocks represented high abundance under group information line.

All samples contained a total of 60 V and 14 J gene segments. In the TRBV3-1, TRBV5-3, TRBV5-7, TRBV7-2, TRBV12-4, TRBV12-5, TRBV24-1, and TRBV30 segments, the LN metastasis group showed significantly higher abundance compared to the control group (**Figure 3A**). The LN metastasis group did not exhibit a significant increase in the abundance of TRB-J segments (**Figure 3B**). Most VJ combinations coexisted in both groups, but there were 54 VJ combinations unique to the metastasis group (**Figure 3C**). These VJ combinations may serve as biomarkers of LN metastasis.

### Specificity of CDR3 sequences in LN metastasis CRC patients

We examined and compared CDR3 sequence abundance between the LN metastasis and control groups. We identified 1,340 upregulated and 1 downregulated differentially expressed amino acid clonotypes (**Figure 3D**). Furthermore, 11 amino acid clonotypes with differential expression were discovered in at least five samples, demonstrating higher abundances in LN metastasis samples (**Figure 3E**). These sequences have the potential to identify LN metastasis in tumor tissues.

### Prediction model of LN metastasis in CRC patients

**Figure 4:**
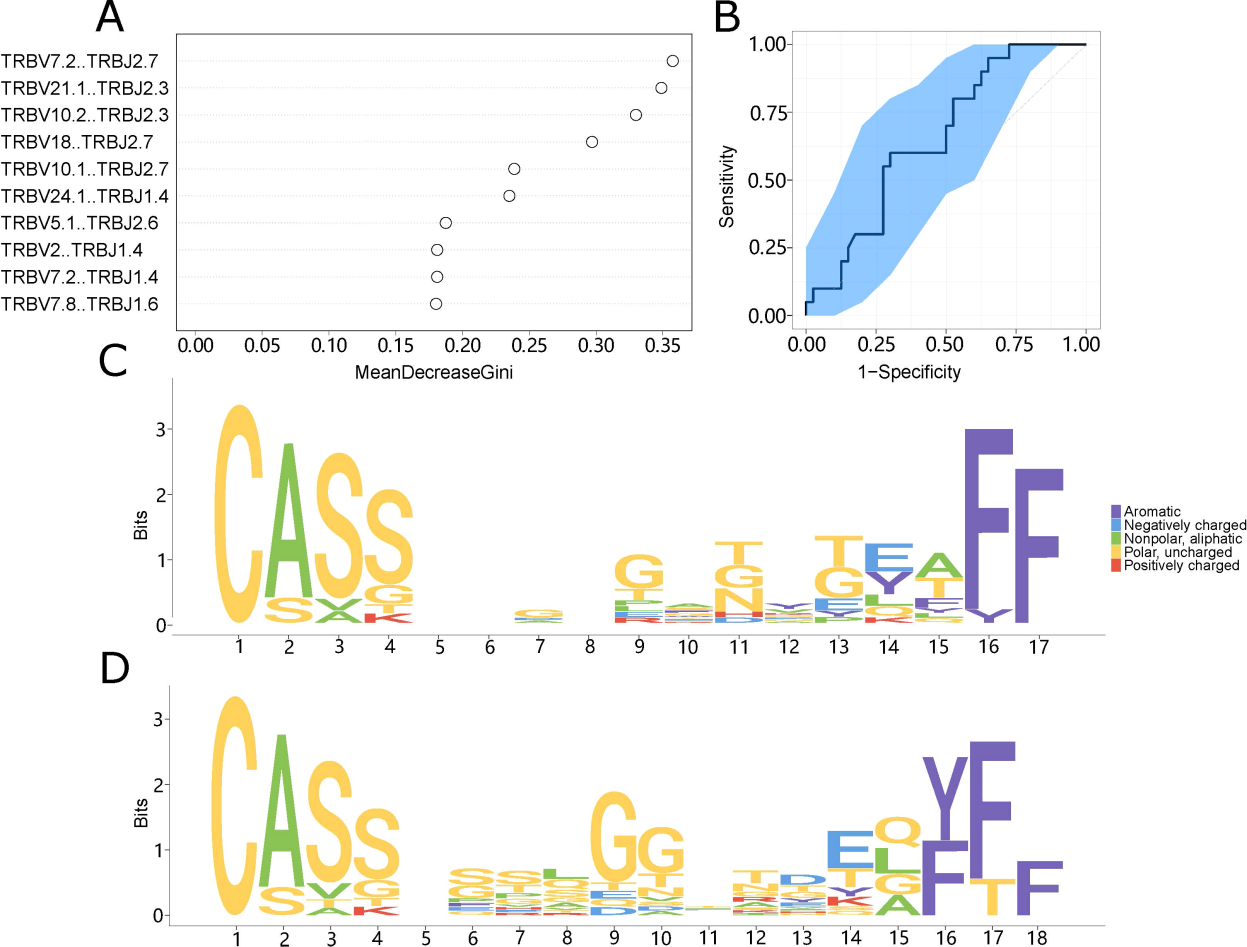
The prediction model system. **(A)** The top ten V-J combinations influencing model impact. Mean decline Gini is a rating index whose value is proportional to the degree of influence on the model. **(B)** The model’s impact on categorization is indicated by the ROC curve. The abscissa indicates the false positive rate (1-specificity), while the ordinate value indicates the real positive rate (sensitivity). **(C, D)** Motif specificity of the TCR repertoires of the LN metastasis **(C)** and control **(D)** groups.

Based on variations in TCR repertoire characteristics, we developed a prediction model utilizing a random forest method to predict LN metastasis in colorectal cancer patients. We imported all the differentiated V-J combinations into the random forest model as judgment vectors and designed the judgment metrics for the classification predictor based on the expression abundance of V-J combinations which appeared in different patients. We adjusted the settings from 0.2 to 0.3 to enhance the model’s precision. After this adjustment, the classification function stabilized. We assessed which V-J combinations influenced the model’s assessment and identified the top 10 combinations (**Figure 4A**) that demonstrated the largest contributions. The model could clearly distinguish between patients with LN metastasis and those without (AUC = 0.514-0.794), and the distribution of the ROC curve was relatively flat (**Figure 4B**).

### Motif analysis of LN metastasis in patients with CRC

To create a motif diagram, we aligned CDR3 sequences present in at least five samples according to their length. These motifs often have functional significance and could represent specific domains in LN metastasis and control patients. This analysis revealed conserved sequences that play important roles in LN metastasis. There were clear differences between the motifs of the LN metastasis and non-LN metastasis groups (**Figures 4C, D**), suggesting that choosing an appropriate amino acid sequence could help identify the specific type of LN metastasis present.

## Discussion

The tumor immune microenvironment (TIME) is highly complex. While the host immune system can identify and eliminate tumor cells, tumors adopt various strategies to evade detection by the host immune system (26). Studies have established that the immune repertoires of tumor tissues differ from those of normal tissues or healthy patients (27–30). For example, in hepatocellular carcinoma, immune repertoire features have been found to differ between tumorous and non-tumorous tissues. Several marker genes of activated T cells have been identified that exhibit high expression levels in tumor tissue (27). Similarly, another analysis revealed multiple dimensions of differences between lung cancer patients and healthy control samples (31). These results are useful for developing potential prognostic biomarkers of the TCR repertoire (31, 32).

In many solid tumors, lymph nodes (LNs) are frequently the first sites of cancer metastasis, and accumulating data have shown that the LN microenvironment provides a favorable environment for cancer cell seeding and proliferation. LN-resident immune and stromal cells are known to influence the microenvironment (33). The draining LNs are in closest proximity to tumor tissues and often serve as the principal sites of tumor metastasis. Suppressing the antitumor immune response within these LNs significantly reduces barriers to tumor cell migration. Both inhibitory immune cells in the TIME and suppressive cytokines released by the tumor can actively spread to nearby LNs. Consequently, this condition affects the functioning of T lymphocytes within draining LNs. T-cell activation in these nodes directly influences the organism’s ability to mount a powerful antitumor response (34). Previous studies have shown that metastatic LNs can alter tumor immune responses, and targeting metastatic LNs can greatly enhance the therapeutic effect on primary cancers (35).

The characterization of the immune repertoire and its clinical significance for LN metastasis in CRC tumor tissues remain poorly understood. In this study, we analyzed the immune repertoires of CRC patients with LN metastasis to demonstrate the relationship between immunity and LN metastasis. We performed TCR sequencing in both LN metastasis and control CRC patients to investigate characteristics related to specific immunity during LN metastasis. Our goals were to elucidate TCR characteristics and identify potential immune markers for the diagnosis and management of LN metastasis.

We found that immune repertoire diversity was lower in CRC patients than in healthy samples. CRC patients exhibited much lower immune system diversity and fewer immune clonotypes than healthy samples, consistent with previous studies of other tumors (27, 36–38). Regardless of LN metastases, the immune system undergoes dramatic changes compared to healthy samples. LN metastasis activated portions of the immune response, resulting in a more favorable immune system profile for LN metastasis samples than for control samples. Despite differences in clonotype numbers and diversity of the immune repertoire between the LN metastasis and control groups, the general framework of VJ gene combinations remained similar between these two groups. These findings suggest that although there are significant differences between the LN metastasis group and the control group, LN metastasis did not fundamentally alter the immune environment.

Although the V and J gene patterns were similar among these two groups, we identified statistically significant differences in the abundance of specific clonotypes, indicating that LN metastasis may lead to the expansion of certain immune responses while constraining others. This highlights the complex interplay between tumor progression and the immune response, suggesting potential avenues for therapeutic intervention aimed at modulating immune activity in the context of LN metastasis. These V-J combinations or immune CDR3 sequences that appeared specifically in LN metastasis group indicated that specific changes in the immune system occurs during the LN metastasis. This phenomenon possibly due to the immune response after the occurrence of LN metastasis. These also explains the overall higher level of immunity after LN metastasis. Differential V-J and CDR3 sequences in TCRs of LN and non-LN metastasis patients may predict LN metastasis in CRC. Though our predictive classifier shows promise, more samples are needed to validate its clinical utility.

By visualizing the abundance of immune CDR3 sequences in the LN metastasis and control groups, we validated our findings in patients with and without LN metastasis. Our results showed that the immune repertoire characteristics in patients with and without LN metastasis may differ. Comparisons of immune repertoires revealed higher quantities of clonotypes in both immune V-J combinations and CDR3 sequences in LN metastasis samples. This finding was also supported by metastatic immune analyses from other tumor studies (39–41). We found that the diversity of the immune repertoire in the LN metastasis group was more complex than that in the control group. PCA clustering highlighted that immune clonotypes were able to discriminate between the LN metastasis and control groups. These findings indicate that LN metastasis initiated several immune clonotypes as an immune defense response to metastasis. These immune clonotypes were either absent or were infrequently present in the control group. Previous studies have shown similar results in other tumors (42, 43), suggesting that LN metastasis affects the complexity of the immune system in CRC patients.

This study has several limitations. All samples were collected from one center, and the sample size was modest. This analysis was based on bulk tissue samples, which could not identify specific immunological cells. Finally, the specific function of each immune clonotype could not be determined. Therefore, large-scale investigations in the future are warranted.

In conclusion, we used high-throughput sequencing of the immune repertoire to demonstrate the characteristics of TCR repertoires in CRC patients with LN metastasis. Our findings suggest that specific TCR clonotypes are potential biomarkers for CRC diagnosis and other clinical scenarios. This study may serve as a foundation for further research on particular TCR clonotypes.

## Data Availability

The datasets presented in this study are deposited in the NCBI repository, accession number PRJNA1049886.
